# Pseudomembranous cystitis in cats with presumed or confirmed mineralization: A retrospective study of 26 cases (2016‐2021)

**DOI:** 10.1111/jvim.16819

**Published:** 2023-07-27

**Authors:** Olivier Labelle, Dominique Penninck, Emmanuelle M. Butty, Shelly Hahn, Marilyn Dunn

**Affiliations:** ^1^ Veterinary Teaching Hospital of the University of Montreal (CHUV) St. Hyacinthe Quebec Canada; ^2^ Tufts Cummings School of Veterinary Medicine North Grafton Massachusetts USA; ^3^ NAMSA Minneapolis Minnesota USA

**Keywords:** Corynebacterium, encrusting, ultrasonography, urethral obstruction

## Abstract

**Background:**

Pseudomembranous cystitis (PMC) in cats is a recognized disease, but concurrent mineralization is reported rarely and its outcome is poorly described.

**Hypothesis and Objectives:**

Describe a population of cats with PMC and the prevalence of concurrent mineralization.

**Animals:**

Twenty‐six cats with PMC.

**Methods:**

Medical records were retrospectively reviewed (January 2016 to December 2021). Cats with an ultrasound diagnosis of PMC were included. Clinicopathologic results, imaging, treatment, and outcome were reviewed.

**Results:**

All cats were male and 21 (80%) were diagnosed with urethral obstruction (UO). Five cats (23.8%) had positive urine culture (*Staphylococcus felis*, 3/5; *Proteus mirabilis*, 2/5) with a median urine pH of 8 (range, 6‐9). All cats had ultrasonographic changes suggestive of mineralization. On ultrasound examination, 10 cats (38.5%) had pseudomembranes with acoustic shadowing suggestive of mineralization, 15 (57.7%) had changes indicative of ulceration, and 8 (31%) had changes compatible with of a urachal anomaly. Twenty‐two cats received medical treatment, 4 underwent surgery (3 percutaneous cystolithotomy, 1 cystotomy). Twenty cats (77%) survived to discharge. Follow‐up ultrasound examination indicated resolution of PMC in 6/7 cats, 4 had persistent hyperechoic bladder lining. Five of 12 cats with follow‐up had a relapse of lower urinary tract signs.

**Conclusions and Clinical Importance:**

Pseudomembranous cystitis was diagnosed mainly in male cats with UO and imaging findings suggestive of mineralization were present in all cases. Frequent negative urine culture suggests a different etiology than encrusting cystitis related to urease‐positive bacteria. Good outcomes can be achieved with medical management.

**Abbreviations**
AR: abdominal radiograph
BCS: body condition score
CKD: chronic kidney disease
EC: encrusting cystitis
FIC: feline idiopathic cystitis
FISH: fluorescent in situ hybridization
PCCL: percutaneous cystolithotomy
PMC: pseudomembranous cystitis
UO: urethral obstruction
US: ultrasound
USG: urinary specific gravity
UTI: urinary tract infection
UVJ: uretero‐vesical junction

## 1 INTRODUCTION

Pseudomembranous cystitis (PMC) is a clinical entity that has attracted interest in the past decade but is only reported in a few dogs and cats.^1^, ^2^, ^3^ It is defined as the presence of false membranes consisting of a layer of exudate (necrotic cell detritus, fibrin, blood, inflammatory cells) on a hemorrhagic and ulcerated bladder mucosal surface.^4^ Previous studies have described its unique ultrasonographic findings with complete or partial bladder luminal septations adhering to the bladder wall and sometimes causing compartmentalization of the bladder.^3^, ^5^, ^6^ Histopathology identified mucosal ulceration, necrosis, and presence of intraluminal fibrinosuppurative, necrotic, and hemorrhagic material.^1^, ^3^, ^7^ In male cats, it has been associated with urethral obstruction (UO).^1^, ^2^, ^3^, ^6^, ^7^ Previous veterinary literature has focused on the outcome with surgical and medical management.^1^, ^2^, ^3^ Resolution of the septations has been reported with medical management alone and documented on abdominal ultrasonography.^1^, ^3^ Radiographic, ultrasonographic and histopathologic evidence of concurrent mineralization has been described in some cats with PMC.^1^, ^2^, ^3^, ^7^

Encrusting cystitis (EC) is the most commonly described form of mineralized cystitis and frequently is associated with *Corynebacterium urealyticum* infection.^8^, ^9^, ^10^, ^11^, ^12^ Other urease‐positive bacteria also have been reported.^13^, ^14^, ^15^ Production of urease by these bacteria increases urine pH, resulting in dystrophic mineralization of the damaged bladder wall usually with struvite (ammonium magnesium phosphate).^8^, ^9^

Feline idiopathic cystitis (FIC) is a common disease in the feline population characterized by neurogenic bladder inflammation.^16^, ^17^, ^18^ Male cats affected by FIC with episodes of urethral obstruction (UO) have a 60% likelihood of FIC relapse and a 36% likelihood of re‐obstruction, placing this feline population at increased risk of repeated urogenital interventions.^18^, ^19^, ^20^

Considering that PMC has only been described in cats with UO at increased risk of repeated urological interventions, that mineralization has been only sporadically mentioned and that PMC has been associated with bacterial infection in human and veterinary patients,^1^, ^2^, ^4^, ^18^, ^19^, ^21^ further evaluation of the clinical characteristics of affected cats is needed.

Our objectives were to describe and report the prevalence of the clinicopathologic anomalies, imaging findings, treatment, and outcome of cats with PMC. We hypothesized that PMC with concurrent mineralization is common and that good outcomes can be achieved with medical management.

## 2 MATERIALS AND METHODS

### 2.1 Case selection

The electronic medical records of client‐owned cats with PMC presented to the University of Montreal and Cummings School of Veterinary Medicine at Tufts University between January 2016 and December 2021 were retrospectively reviewed. All cats with an ultrasonographic diagnosis of PMC, based on the presence of intraluminal pseudomembranes, were included. Cases were excluded if the medical records were incomplete.

### 2.2 Medical record review

Signalment (breed, age, sex, neuter status) and clinicopathologic findings including presenting complaint, duration of clinical signs, history of previous urological disorders and interventions, CBC, serum biochemistry, urinalysis, and urine culture and sensitivity results on admission were collected. Azotemia was defined according to International Renal Interest Society (IRIS) guidelines.^22^ Hyperkalemia was defined as >4.2 mmol/L and ionized hypocalcemia as <1.17 mmol/L. Pyuria was defined as >5 WBC/hpf on urine sediment analysis from cystocentesis and catheter obtained samples and >8 WBC/hpf on voided samples. Hematuria was defined as >5 RBC/hpf on voided and catheter‐obtained samples and >20 RBC/hpf on cystocentesis samples. Type of antimicrobial (if administered), time of treatment (before or after urine collection), and sensitivity results were collected. Cystotomy and percutaneous cystolithotomy (PCCL) findings were recorded when performed. Referring veterinarians were contacted for previous medical history of lower urinary tract disorders, for potential relapse of lower urinary tract signs after discharge and for outcome. Relapse of urinary tract disorders was further characterized by the type of urinary tract signs, number of relapses of UO, time between discharge and relapse and presence of positive urine culture. Outcome was reported as dead or alive at the time of writing and cause of death was divided into related or not related to a urinary tract disorder. The time between diagnosis and death or last follow‐up available also was recorded.

Pseudomembranous cystitis was defined as the presence of echogenic septa in the bladder lumen on abdominal ultrasonography. Presumed mineralization was defined on ultrasonography as presence of a hyperechoic interface, with or without acoustic shadowing, seen along the luminal surface of the bladder wall in nondependent or dependent areas. This characterization included changes associated with the bladder wall (eg, adherent or within the wall) and luminal echogenic septa. Confirmed mineralization was defined as amorphous mineralization within the bladder lumen on abdominal radiographs or by endoscopic or surgical visualization of luminal surface crystalline deposits, adhered to intraluminal strands or histopathologic evidence of luminal mineralization. Cystoliths, urethroliths, and ureteroliths were described separately.

### 2.3 Imaging

Abdominal radiographs (AR) as well as ultrasonographic images and videos (US) of the urinary system (urethra, bladder, ureters, and kidneys) were reviewed by a board‐certified radiologist (Penninck).

#### 2.3.1 Abdominal radiographs

The following findings were collected: quality of the radiographic study, degree of distension (classified as mild, moderate, or marked), bladder shape (defined as round, pear‐shaped, or with a pointed apex), presence of confirmed mineralization, presence of cystoliths (number and size), and presence of ureteroliths. Evaluation of the kidneys included subjective size (defined as decreased, increased, or normal), shape (defined as regular or irregular), and presence of nephroliths.

#### 2.3.2 Abdominal ultrasonography

All cats were scanned in dorsal recumbency. The following bladder findings were collected: degree of distension (subjectively classified as mild, moderate, or marked), bladder wall thickening, presence of presumed mineralization with or without acoustic shadowing, urethral thickening, presence of cystoliths, pointed bladder apex (urachal anomalies), presence of perivesical hyperechoic fat or effusion, and presence of ulceration. The latter was defined as defects in the bladder mucosal lining. The pseudomembranes were defined as complete if both ends of the pseudomembranes were attached to the bladder wall and incomplete if only a single end was attached to the bladder wall. The location of the pseudomembranes was described as apical, near the trigone, or within the proximal urethra. The following renal findings were collected: presence of perirenal hyperechoic fat, retroperitoneal effusion, pyelectasia (>1 mm), renomegaly (kidney length >4.5 cm), renal pelvic mineralization, or nephroliths.^23^ Ultrasonographic evidence of chronic kidney disease (CKD) was defined as a combination of ≥2 of the following: irregular cortical margins, decreased corticomedullary distinction, and decreased kidney size (kidney length <3 cm).^23^ Ureters were evaluated for dilatation (visible lumen), wall thickening, and intraluminal content.

### 2.4 Surgical intervention

Percutaneous cystolithotomy (PCCL) and routine cystotomy were performed as previously described. Images of cystoscopy and PCCL were reviewed by 3 small animal board‐certified internists and a small animal internal medicine resident (Butty, Dunn, Labelle). Cystoscopic lesions were characterized based on the severity (mild, moderate, severe) of the following features: confirmed mineralization, ulceration, pseudomembranes, and fibrosis. A bladder wall biopsy was performed with endoscopic biopsy forceps (5 Fr, length 34 cm, biopsy forceps, Karl Storz Endoskope) in all cases that underwent PCCL. The bladder wall biopsy specimens were sent for culture in all PCCL cases. Histopathology and adhered mineralized debris analysis were performed at the discretion of the clinician.^24^, ^25^, ^26^

### 2.5 Histopathology

Histopathology findings were reviewed by a board‐certified pathologist (Hahn) with targeted descriptions of ulceration, hemorrhage, mineralization, and presence of bacteria.

## 3 RESULTS

### 3.1 Clinicopathologic findings

Twenty‐six cats met the inclusion criteria (19 Cummings School of Veterinary Medicine at Tufts University, 7 University of Montreal). All cats were male (23 neutered, 3 intact) with a median age of 5 years (range, 1‐14 years). Mean body weight was 5.7 kg (range, 3.1‐10.1 kg) and median body condition score (BCS) in 23/26 cats was 6/9 (range, 2‐9). Twenty‐one (80%) cats were presented for UO, 3 (11.5%) were presented for hematuria, of which 1 experienced obstruction during hospitalization, and 1 (4%) was presented for suspected urethral stricture. Median duration of clinical signs was 2 days (range, 1‐58 days). Thirteen of the 23 cats with a medical history available had previous lower urinary tract signs. Of these 13, 7 had a history of UO, 5 had suspected episodes of FIC without obstruction, 1 had a history of urinary incontinence and 1 had a history of urinary tract infection (UTI). Eight (31%) cats had a history of urinary tract catheterization that occurred between 21 days and 3 years before presentation. Seven (27%) cats received prior antibiotic therapy, a mean of 28 days before presentation (range, 1‐78 days).

On presentation, 25 (96%) cats were azotemic with a mean serum creatinine concentration of 6.1 mg/dL (range, 1.55‐14.9 mg/dL). One cat had a serum creatinine concentration too high to measure and was censored. Mean blood urea in 23/26 cats was 100 mg/dL (range, 25.23‐206 mg/dL), with 8 results too high to determine and censored. Nineteen (73%) cats were hyperkalemic with a median serum potassium concentration of 5.4 mmol/L (range, 3.4‐10.3 mmol/L). Sixteen of 24 cats with serum ionized calcium concentration measured were hypocalcemic with a mean concentration of 1.06 mmol/L (range, 0.77‐1.35 mmol/L).

Urinalysis was available in 25 cats. The mean urine specific gravity (USG) was 1.019 (range, 1.009‐1.061) with urine sampled at time of admission in 23 cats, of which 8 had bladder irrigation performed before sampling and 2 that were sampled after fluid therapy. Mean urine pH was 7 (range, 6‐9). All cats had hematuria. Pyuria was identified in 13 (52%) cats and bacteriuria was identified in 6 (24%) cats (samples via voiding [2], catheter [2] and cystocentesis [2]). Struvite crystals were identified in 8 (32%) samples and calcium oxalate crystals in 1 sample. Twenty‐one (81%) cats had a urine culture performed and 18 (86%) were submitted before antibiotic administration. Five cats (24%) had positive cultures with a single organism. The isolated bacteria were *Staphylococcus felis* in 3 cats and *Proteus mirabilis* in 2 cats. One sample was positive with a mixed flora and <10 000 cfu/mL on a catheter‐obtained sample, which was deemed to represent contamination. In the 5 cats with positive cultures, mean urine pH was 8 (range, 6‐9). Two cats had positive urine cultures with pyuria but no bacteriuria. One cat had a positive urine culture with no urine sediment available for review.

Twenty‐five cats had an indwelling catheter placed and 22 (85%) were medically managed using buprenorphine (0.01‐0.02 mg/kg IV or PO q6‐8h), gabapentin (5‐22 mg/kg PO q8‐12h), methadone (0.1‐0.2 mg/kg IV q4‐6h), prazosin (0.5 mg/cat PO q8‐12h), tamsulosin (0.07 mg/kg PO q24h), meloxicam (0.03‐0.1 mg/kg IV or PO q24h), robenacoxib (2 mg/kg SC q24h), prednisolone (0.22‐0.66 mg/kg PO q24h), bethanechol (1.3 mg/kg PO q24h), fluids and supportive care (maropitant, 0.72‐1 mg/kg PO or IV q24h; ondansetron, 0.5 mg/kg IV q12h; mirtazapine, 1.87‐3.75 mg/cat PO q48‐72 h; pantoprazole, 1 mg/kg IV q12h) or some combination of these. Antibiotics were administered in 19 (73%) cats. Sixteen (84%) cats received ampicillin (22‐30 mg/kg IV q8‐12h), ampicillin/sulbactam (30‐35 mg/kg IV q8h) or amoxicillin/clavulanate (10.9‐20 mg/kg PO q12h). Eight cats received enrofloxacin (2.2‐5 mg/kg IV/PO q12‐24 h) or marbofloxacin (3.4‐5.6 mg/kg PO q24h). One cat received cefazolin (22 mg/kg IV q8h).

### 3.2 Abdominal radiographs

Twenty‐five cats had abdominal radiographs (AR) available for review. The quality of AR was defined as excellent in 6 cats, good in 11 cats, adequate in 3 cats, and poor in 5 cats. Confirmed mineralization was noted in 10 (40%) cats. Bladder shape could be assessed in 19/25 cats. Radiographic changes with apical bladder wall elongation (pointed apex) was noted in 4/19 cats. Radiopaque cystoliths were identified in 3 (12%) cats, of which 1 had confirmed mineralization on AR. No nephroliths or ureteroliths were identified.

### 3.3 Abdominal ultrasonography

All cats with PMC had hyperechoic changes supportive of presumed mineralization (Table 1). Ten (38%) cats had pseudomembranes with shadowing, suggestive of pseudomembrane mineralization. Mean pseudomembrane thickness was 1.47 mm (range, 0.3‐4.9 mm). Twenty (77%) cats had complete and 6 (23%) had partial pseudomembranes. Six (23%) pseudomembranes had a lacy appearance, 1 was causing compartmentalization of the bladder, 3 were attached near the trigone and uretero‐vesical junction (UVJ), and 2 were identified within the proximal urethra.

**TABLE 1:** Abdominal ultrasound findings.

Findings	Number (total)	(%)	Median size (mm)	Range (mm)
Pseudomembranes	26	100	1.47	0.3‐4.9
	Complete (20)	77		
	Partial (6)	23		
Hyperechoic lining	26	100	0.7	0.2‐1.3
	Diffuse (24)	92		
	Discontinuous (2)	8		
Bladder wall thickening	Diffuse (25)	96	Ventral 7.5	1.3‐13.6
			Dorsal: 4.3	1.7‐7.7
	Layered thickening (21)	81		
	Urachal remnant (8)	31		
Cystolith	5	19	3.9	1‐9.3
Kidneys	Renal evaluation (25 cats)		45	38‐53
	Renomegaly (16 cats)	62	48	45‐53
	Pyelectasia (13 cats)	53	1.90	1.1‐11
Ureter	Ureteral dilatation (10 cats)	38	2.5	1.0‐4.6
	Unilateral dilatation (5 cats)	50		
	Bilateral dilatation (5 cats)	50		
Urethra	17			
	Urethral thickness	—	1.5	1.2‐3.4
	Urethral dilatation	18	4.1	3.3‐5.2
	Urethral pseudomembrane	12		

**FIGURE 1:**
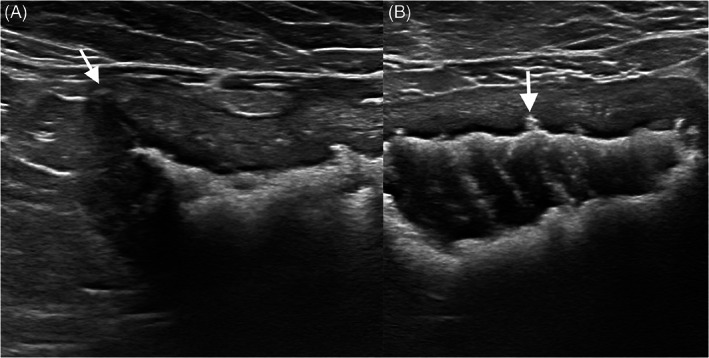
Longitudinal sonogram of the bladder of an 8‐year‐old castrated male domestic medium hair cat with PMC and mineralization. (A) The apex of the bladder was pointed (arrow). The bladder wall reached up to 6.5 mm in thickness. Mineralized (confirmed radiographically) lining associated with shadowing was noted along the ventral bladder wall. (B) Transverse sonogram after filling the bladder with saline. Numerous hyperechoic strands are seen crossing the lumen. Mucosal ulceration (arrow) is noted at several locations along the ventral wall.

All cats had either diffuse (24) or regional (2) hyperechoic mucosal bladder lining compatible with presumed mineralization. The mean hyperechoic lining thickness was 0.7 mm (range, 0.2‐1.3 mm). Ultrasonographic findings suggestive of bladder wall erosion or ulceration were noted in 15 (58%) cats (Figure 1).

**FIGURE 2:**
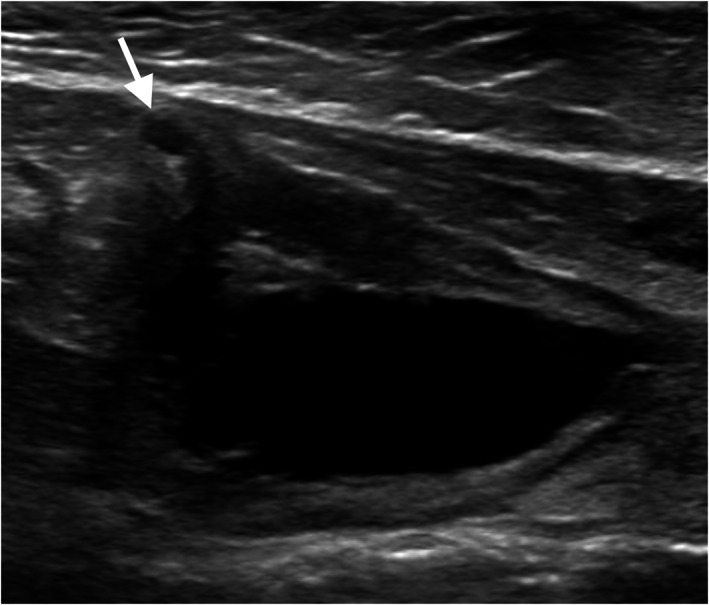
Longitudinal sonogram of the thickened (6 mm) ventral bladder wall of a 7‐year‐old castrated male domestic short hair cat with incomplete membranes and partially hyperechoic mucosal lining. An anechoic track was dissecting the cranioventral wall, leading to a small ampullar diverticulum (arrows) which likely represents a urachal remnant. No mineralization was seen radiographically.

All cats had bladder wall thickening with a mean ventral wall thickness of 7.5 mm (range, 1.3‐13.6 mm) and mean dorsal wall thickness of 4.3 mm (range, 1.7‐7.7 mm). One cat had focal ventral thickening whereas all others had diffuse bladder wall thickening. In 21 (81%) cats, the bladder wall thickening had a layered appearance, with both hyperechoic and hypoechoic layers. Findings supportive of a urachal anomaly (cystic diverticulum at the apex or pointed apex) were detected in 8 (31%) cats, resulting in focal bladder wall thinning at the apex in 7 cats (Figure 2). The bladder wall was described as undulating in 9 cats. Uroliths were identified in 5 (19%) cats. Mean urolith size was 3.9 mm (range, 1‐9.3 mm). Perivesical effusion and hyperechoic fat were noted in 15 (58%) and 19 (73%) cats, respectively. Two cats required retrograde saline infusion to identify pseudomembranes (1) and urachal anomaly (1).

Ultrasonographic images of the kidneys were available in 25/26 cats. Renomegaly was noted in 15 cats (11 bilateral, 5 unilateral). Median kidney length was 4.5 cm (range, 3.8‐5.3 cm). Ultrasonographic changes suggestive of CKD were detected in 2 cats. Pyelectasia was present in 13 cats (7 unilateral, 6 bilateral) with a mean renal pelvic height of 1.9 mm (range, 1.1‐11 mm) and renal pelvic recess dilatation was present in 3 cats (2 bilateral, 1 unilateral). Medullary striations were identified in 2 cats (2 bilateral). One kidney had a hyperechoic lining of the renal pelvic cavity suggestive of encrusting pyelitis or chronic pyelitis or pyelonephritis. Ureteral dilatation was noted in 10 cats (5 bilateral, 5 unilateral) and was proximal in 8, distal in 2, and diffuse in 5 ureters. Mean ureteral dilatation was 2.5 mm (range, 1.0‐4.6 mm). Other ultrasonographic findings are reported in the Table S1.

**FIGURE 3:**
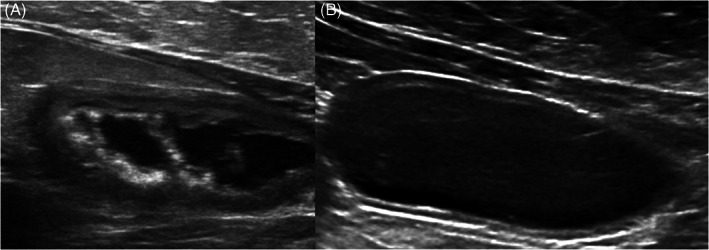
Longitudinal sonogram of the thickened bladder (5.2 mm) of a 5‐year‐old castrated male domestic medium hair cat with PMC and mineralization. (A) Hyperechoic membranes and mineralized lining covering part of the mucosa were noted. (B) Repeat ultrasonography 2 months later showed resolution of the pseudomembranes and bladder wall thickness returned to normal (2 mm).

Follow‐up ultrasonography was performed in 7 cats. Median follow‐up time was 15 days from discharge (range, 10‐57 days). Resolution of pseudomembranes was noted in 6/7 (85%) cases (Figure 3). Persistent wall thickening and hyperechoic mucosal lining compatible with presumed mineralization were noted in 3 cats. One cat had persistent hyperechoic mucosal lining only and 1 had persistent bladder wall thickening only. One cat with ureteral dilatation and conservative medical management only had resolution of the dilatation on follow‐up ultrasonography.

### 3.4 Surgical interventions

Surgery was performed in 4 (15%) cats. One cat underwent routine cystotomy for urolith removal. The bladder wall was severely thickened and turgid with irregular serosa. The bladder lumen was filled with small irregular calculi and the bladder wall had multifocal areas of adhered mineralization which was debrided. The bladder lumen was filled with blood clots and a mineralized pseudomembrane also was debrided from the bladder apex. Urolith analysis identified 100% calcium oxalate. Mineral debris associated with the pseudomembrane was not analyzed.

**FIGURE 4:**
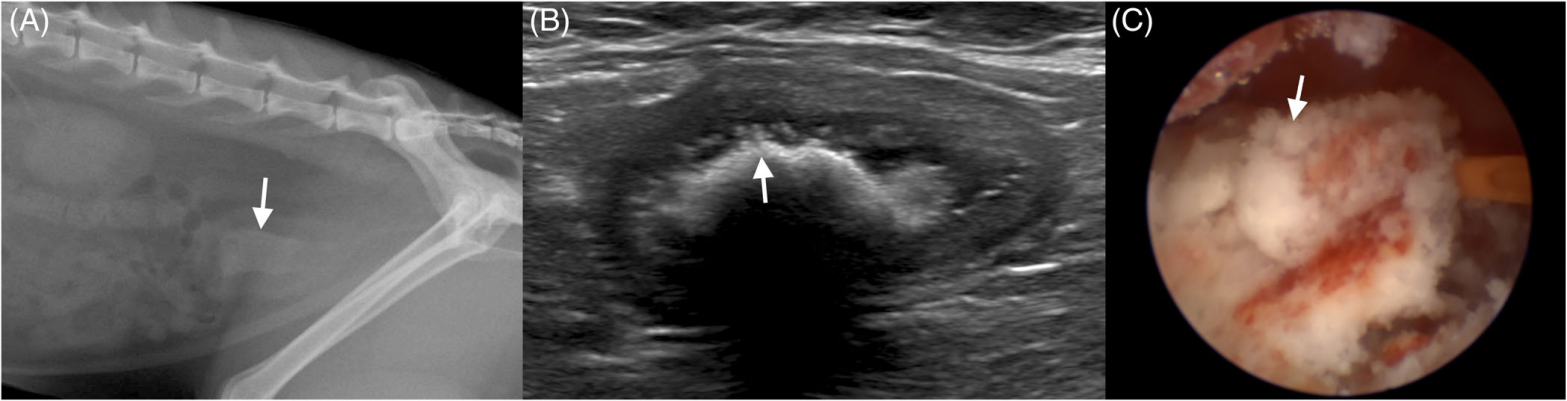
Abdominal radiograph, sonogram, and cystoscopic image of a 5‐year‐old castrated male domestic long hair cat with PMC and mineralization. (A) On the abdominal radiograph, an elongated radiopacity outlining the bladder lumen was observed (arrow). (B) On abdominal ultrasonography a hyperechoic pseudomembrane with acoustic shadowing was identified (arrow). Shallow indentations along the ventral mucosal surface represent superficial ulcerations. (C) On cystoscopy, the bladder surface was covered with mineral debris (arrow) and mineralized pseudomembranes are seen.

Three cats underwent PCCL and had endoscopic evidence of confirmed mineralization (Figure 4). One cat had PCCL performed because of suspected uroliths on ultrasonography. Two cats had PCCL performed at the same time as a perineal urethrostomy because of recurrent UO suspected to be secondary to PMC and concurrent mineralization. One cat had no pseudomembranes reported on endoscopic examination. This cat had complete pseudomembranes on US 3 days before PCCL. Mineralization and pseudomembranes were debrided using an endoscopic retrieval basket (1.9‐Fr stone basket DAKOTA Nitinol Stone Retrieval Device 1.9 Fr × 120 cm 11 mm; Boston Scientific, Spencer, Indiana). All cats had evidence of bladder ulceration after debridement. No macroscopic evidence of fibrosis was noted. No cats had bacterial growth on bladder wall culture, but all received antibiotics before surgery with ampicillin/sulbactam (30 mg/kg IV q8h) or enrofloxacin (5 mg/kg IV q24h). Mineralized debris was sent for mineral analysis in 1 cat and found to be 100% struvite.

### 3.5 Histopathology

Histopathology of bladder wall biopsy samples was available for review in 6 (23%) cats. Diffuse, severe, chronic suppurative, and fibrinous cystitis was identified in 4 cats. Of these, 2 were also hemorrhagic, 1 had identifiable ulcerations, and 1 had fibrinous vascular thrombosis. Chronic ulcerative cystitis was diagnosed in 1 cat, and mild to moderate chronic hyperplastic cystitis in the other cat. Material excreted during micturition was available for review in 1 cat and consisted of a mixture of mineralized material and collagen. A Brunn's nest was identified in 1 sample. Bladder wall edema was noted in 1 sample. A bladder smooth muscle hamartoma was identified in 1 cat. Intraluminal cocci colonies were identified in 1 cat, but because of the severity of bladder wall necrosis, intralesional cocci could not be excluded.

### 3.6 Outcome

Twenty (77%) cats were alive at the time of discharge. Median hospitalization time was 4 days (range, 2‐8 days). Two cats were euthanized as a result of complications related to fluid overload and 4 cats were euthanized because of owner financial limitations. Follow‐up information was available for 13 (50%) cats. Median follow‐up time was 469 days (range, 37‐1892 days). Five of 13 cats had recurrence of lower urinary tract signs: 4 had pollakiuria, periuria, or both, and 1 had intermittent dysuria. Two cats had recurrent UO: 1 relapsed the day after discharge and the other 84 days after discharge. Of the 13 cats with follow‐up information, 2 were euthanized: 1 because of UO recurrence 87 days postdiagnosis, the other because of open‐mouth breathing 207 days postdiagnosis. The cat with imaging suggestive of encrusting pyelitis or chronic pyelitis or pyelonephritis had a negative urine culture before antibiotic treatment and was azotemic at discharge (serum creatinine concentration, 4.4 mg/dL) and discharged on marbofloxacin. This cat was still azotemic 2 months after discharge (serum creatinine concentration, 2.2 mg/dL). No follow‐up ultrasonography was performed. The cat presented in cardiac arrest 469 days after diagnosis. No necropsy was performed. Ten cats were still alive at the time of writing.

## 4 DISCUSSION

We described a population of cats with an ultrasonographic diagnosis of PMC. All cats were found to have confirmed or presumed mineralization on imaging. All cats were male, most frequently presented with a UO (80%) and negative urine culture (76%).

Although PMC has been described secondary to infection or bladder overdistension in human patients,^4^, ^27^, ^28^, ^29^ the inciting cause of PMC remains unclear in cats. In cases of urethral obstruction, substantial bladder overdistension has been shown to alter bladder vascularization and even result in thrombosis.^28^, ^30^ These changes were identified in 1 biopsy sample in our study. However, PMC is an uncommon diagnosis in cats presenting with UO, and the reason why only a small subpopulation of cats develops these changes remains unknown.

The confirmed or presumed mineralization present in all of our cats may mimic previously described encrusting cystitis (EC).^8^, ^9^, ^10^, ^11^, ^12^, ^13^, ^14^, ^15^, ^31^ The differentiation between EC and PMC with mineralization is important because the initial underlying pathophysiologic mechanism appears to differ. Encrusting cystitis results from urease‐positive bacterial infection causing secondary mineralization of the bladder wall.^8^, ^9^, ^31^ Bacterial infection does not appear to be an important contributing factor in PMC with concurrent mineralization, based on our study and a recent report.^3^ Bacterial infection was not commonly identified in our cat population (5/21; 24%), which is similar to the rate of bacterial UTI reported in FIC.^20^, ^32^, ^33^, ^34^ It is considered unlikely that *C. urealyticum* infection was missed in our samples because all bacterial cultures were held for at least 48 hours, the minimal incubation time required for *C. urealyticum* growth, and most (86%) cultures were performed before antibiotic treatment.^9^, ^31^, ^35^ However, not all cultures were held for 72 hours, therefore *C. urealyticum* infection cannot be conclusively ruled out. Bladder wall biopsy identified intraluminal cocci in 1 sample, which differs from the diphtheroid morphology of *C. urealyticum*, but fluorescent in situ hybridization (FISH) was not performed.^31^

Despite the high rate of negative urine culture in PMC, most of the cats in our study received antibiotics.^3^ The rationale behind antimicrobial prescription cannot be ascertained from our study because of its retrospective nature. Antibiotic treatment probably was initiated for suspected bacterial cystitis, while awaiting urinalysis, urine culture, and sensitivity, or both. Our findings suggest that antimicrobial treatment may not be necessary and could be withheld pending urine culture results.^36^ Because the etiology of PMC with mineralization remains elusive, search for intralesional bacteria using FISH and keeping urine cultures for ≥72 hours may help further characterize the role of *C. urealyticum* in the pathogenesis of PMC with concurrent mineralization.

Despite the potential risk of re‐obstruction from mineralized or necrotic debris associated with PMC, most of the cats in our study had resolution of pseudomembranes with conservative medical management only. Despite these severe bladder changes, survival and recurrence rate of urinary tract signs were similar to what has been reported previously for FIC.^19^

Ultrasonographic findings in our study are similar to those previously described with presence of intraluminal pseudomembranes and partial to complete adhesion of pseudomembranes to the bladder wall.^1^, ^2^, ^3^, ^6^, ^7^ The previously described classification scheme was not used in our population because the retrospective nature of the study limited completely reliable evaluation of compartmentalization by the pseudomembranes.^3^ Indeed, 6 cats had retrograde intravesical saline infusion during the ultrasonography study and the bladder filled uniformly, ruling out complete compartmentalization. Only 1 cat had ultrasonographic evidence of bladder compartmentalization on ultrasonography. All cats in our study had ultrasonographic findings supportive of presumed mineralization with presence of a hyperechoic mucosal lining, as reported in EC.^9^ The presence of acoustic shadowing often is a sign of mineralization, but it can sometimes be challenging to see such artifact when the bladder contains a moderate amount of urine. Abdominal radiographs can assist in confirming the presence of mineralization, but the presence of superimposed artifacts (eg, wet hair) may limit the value of radiographs. Other changes to consider are bladder wall fibrosis or adhered fibrinous material. Ten cats (40%) in our study had mineralization confirmed by AR and the 4 cats with surgical intervention had confirmed mineralization. Future observational cohort studies with follow‐up imaging, cystoscopy and bladder wall histopathology would aid in determining whether all cats with PMC have mineralization.

Mucosal erosions seen in 58% of cats appeared as several shallow defects along the bladder wall and are commonly reported in PMC.^1^, ^2^, ^3^, ^7^ Retrograde saline infusion can be useful to further assess bladder defects, the distribution of pseudomembranes and bladder shape, especially if a urinary catheter is present at the time of the US evaluation.

Urachal anomalies were commonly identified in our population (31%). This prevalence is much higher than previously reported and may suggest a potential predisposing factor.^37^ However, the cause‐and‐effect relationship remains unknown, and it could be speculated that bladder overdistention may have stretched the weakest region of the bladder wall, leading to a pointed apex.

As mentioned in a recent report, pyelectasia and ureteral dilatation have been identified in this population.^3^ Pseudomembranes located in the trigone were inconsistently identified in cases of suspected ureteral obstruction. Ureteral dilatation could occur because of obstruction of the UVJ by pseudomembranes, uretero‐vesical reflux because of increased bladder pressure, or mineralization causing decreased bladder compliance or direct UVJ obstruction.^38^ When follow‐up ultrasonography was available, all of these changes had resolved.

Previous studies reported ultrasonographic resolution of pseudomembranes as early as 10 days after diagnosis.^2^, ^3^ Resolution might have been missed in the study population because it can occur at variable rates and was not monitored over time. Resolution of the hyperechoic lining however would not be expected if these changes were caused by fibrosis.

One cat had ultrasonographic evidence of suspected mineralization of the renal pelvis which can be suggestive of encrusting pyelitis.^39^ It cannot be conclusively ruled out that this cat had an occult urease‐positive pyelitis, which could have contributed to its persistent azotemia or death, but the lack of follow‐up imaging makes the clinical relevance of these findings uncertain.

Procedures such as cystotomy or PCCL were attempted for urolith removal in 2 cats and recurrent obstruction in 2 cats. Interestingly, a suspected urolith on ultrasonography was found to be mineralization during PCCL in 1 cat. This finding illustrates that PMC with mineralization can mimic uroliths and should be suspected when the findings are not gravity dependent on ultrasonography. In the 2 cats that had PCCL with recurrent UO, it is uncertain whether pseudomembranes were present and mineral debridement was beneficial because both had perineal urethrostomy surgery performed concurrently.

Histopathology identified fibrous tissue in 3/6 (50%) bladder samples, which also has been reported in cases of FIC.^40^ These findings suggest a more chronic etiology despite the acute presentation in the majority of cases. Because the majority of cats were presented for UO, it can be speculated that repeated bladder distention may result in altered wall contractibility (which may be supported by the undulating appearance of the wall on US in 23% of the cats) and may lead to fibrotic changes.^41^ Ulcerations, Brunn's nest, and bladder wall edema identified in biopsy samples may reflect FIC as the initial cause of clinical signs or may highlight the similarities between these 2 conditions.^40^

Because of its retrospective nature, our study had several limitations. Variations in therapeutic plans and the absence of a control group preclude us from making clear therapeutic recommendations for this population of cats. Furthermore, PMC diagnosis was an ultrasonography‐based diagnosis and the presence of pseudomembranes was not confirmed using other imaging modalities (such as cystoscopy) in all cases. Our study is still relevant because in the feline PMC population, cystoscopic confirmation rarely is performed. The absence of histopathology or cystoscopy in all cases precludes us from confirming mineralization, and the exact location of the mineralization could not be determined in most cats. Another important limitation is the absence of a control group of cats diagnosed with EC caused by urease‐positive bacteria, precluding comparison between the 2 conditions. Lastly, it was at the clinician's discretion to search for specific infectious agents and the absence of FISH and urine cultures held for ≥48 hours prevented us from completely ruling out the presence of *C. urealyticum*.

In conclusion, PMC in cats was associated with presumed or confirmed mineralization of the bladder. This mineralization does not appear to be associated with urease‐positive bacterial infection, suggesting a different pathogenesis than for encrusting cystitis, although *C. urealyticum* cannot be conclusively ruled out and its role in PMC with concurrent mineralization requires further study. Despite the severe bladder changes, the majority of cats responded to medical management.

## CONFLICT OF INTEREST DECLARATION

Authors declare no conflict of interest.

## OFF‐LABEL ANTIMICROBIAL DECLARATION

Authors declare no off‐label use of antimicrobials.

## INSTITUTIONAL ANIMAL CARE AND USE COMMITTEE (IACUC) OR OTHER APPROVAL DECLARATION

Authors declare no IACUC or other approval was needed.

## HUMAN ETHICS APPROVAL DECLARATION

Authors declare human ethics approval was not needed for this study.

## Supporting information


**Table S1.** Additional ultrasonographic findings.
