# Supplementary material for: Pseudomembranous cystitis in cats with presumed or confirmed mineralization: A retrospective study of 26 cases (2016‐2021)
Source: J Vet Intern Med. 2023 Jul 27;37(5):1806–14. doi: 10.1111/jvim.16819 (PMC10472995; doi:10.1111/jvim.16819)
Supplement: Supplementary file 1 — Table S1. Additional ultrasonographic findings. [file JVIM-37-1806-s001.pdf]

Supplementary Table 1 : Additional ultrasonographic findings

		NUMBER
**BLADDER DISTENSION (CATS)**	Collapsed	6
	Mild	13
	Moderate	6
	Severe	1
**RETROPERITONEAL EFFUSION**	Presence (kidneys)	22
**PERIRENAL HYPERECHOIC FAT**	Presence (kidneys)	8
